# Urinary biomarkers in multicentric studies: Shaping the future of bladder cancer diagnosis and follow‐up

**DOI:** 10.1002/bco2.70124

**Published:** 2025-12-18

**Authors:** Alexandrine Martel, Lucas Raue, Patrice Hodonou Avogbe, Jennifer Raisch, Claudio Jeldres, Thorsten Ecke, Emmanuel Vian, Md Ismail Hosen, Anja Rabien, Florence Le Calvez‐Kelm, Francois‐Michel Boisvert

**Affiliations:** ^1^ Department of Immunology and Cell Biology, Faculty of Medicine and Health Sciences Université de Sherbrooke Sherbrooke Canada; ^2^ Urology Universitätsmedizin Berlin Charité Berlin Germany; ^3^ International Agency for Research on Cancer (IARC) Lyon France; ^4^ Division of Urology, Faculty of Medicine and Health Sciences Université de Sherbrooke Sherbrooke Canada; ^5^ Urology Helios Hospital Bad Saarow Germany; ^6^ Urology Department Protestant Clinic of Lyon Lyon France; ^7^ Department of Biochemistry and Molecular Biology University of Dhaka Dhaka Bangladesh

**Keywords:** bladder cancer, cystoscopy alternatives, cytology, genomic, haematuria detection, liquid biopsies, multicentre studies, non‐invasive diagnosis, proteomic, urinary biomarkers, urothelial carcinoma

## Abstract

**Background and Objective:**

Bladder cancer (BC), a prevalent malignancy, poses significant diagnostic and surveillance challenges due to its high recurrence rates and reliance on cystoscopy, an invasive procedure for diagnosis and monitoring. While urine‐based genomic and proteomic biomarkers offer promising non‐invasive alternatives, their clinical implementation remains limited. This review synthesizes evidence from multicentric studies on urinary biomarkers for BC and evaluates their potential in reducing unnecessary invasive cystoscopies.

**Methods:**

A comprehensive review of literature was conducted searching for multicentric studies on urine‐based genomic and proteomic biomarkers for BC detection and/or surveillance. MEDLINE/Pubmed, Embase and Scopus databases and BJUI, UroToday and European Urology Oncology registries were searched using National Library of Medicine Medical Subject Headings (MeSH) terms. Emphasis was placed on the comparative performance of diagnostic platforms across different research and clinical settings.

**Key Findings and Limitations:**

The literature search yielded 51 reports that were included for analysis. Multicentre studies enhance the generalizability of findings by addressing inter‐laboratory variability and population diversity. This review underscores the importance of standardization, comparative performance analyses that these studies provide, and the potential for cost‐effective non‐invasive diagnostic tools. However, despite FDA approvals, no biomarker has replaced cystoscopy in clinical settings due to an inconsistent and insufficient combination of sensitivity, specificity and cost‐effectiveness parameters. The performance of AssureMDX and Enhanced CxBladder tests showed the most promise, but further large‐scale, standardized validation is still necessary.

**Conclusions and Clinical Implications:**

Urine‐based biomarkers have the potential to improve early BC detection and surveillance while reducing reliance on invasive procedures and costs related to the disease. Future efforts should prioritize cost‐effective, large‐scale multicentric studies to facilitate the adoption of these biomarkers into routine practice.

## 1 INTRODUCTION

Bladder cancer (BC) ranks as the 9th most common cancer globally, with approximately 614 000 new cases and 220 000 deaths reported in 2022. It disproportionately affects men (3–4x higher incidence), although women often present with higher‐grade disease and worse outcomes.^1^ Smoking drives 50–65% of cases in men and 20–30% in women, while occupational carcinogens contribute to 5–25% and 8–11%, respectively.^2^ However, disparities persist due to biological factors such as immune differences, hormones and genetic or epigenetic alterations.^3^ BC treatment is complex due to its heterogeneity and high recurrence rates, presenting a significant burden on patients and healthcare systems. Urothelial carcinoma (UC), the most common subtype (90% of BC), includes Non‐Muscle Invasive (NMIBC, 75%) or Muscle‐Invasive (MIBC, 25%). Other subtypes, such as squamous cell carcinoma, adenocarcinomas and rare sarcomas, add to the disease's complexity.^4^

Haematuria is the most common clinical symptom, yet its evaluation is challenging as it often arises from non‐malignant conditions. Visible haematuria is associated with a higher prevalence of BC (17–20%) compared to non‐visible haematuria (0.4–6.5%). Timely evaluation and referral are essential for early detection of BC.^5^ Recent updates to the American Urological Association (AUA) Microhaematuria Guideline provide a refined framework for risk stratification and patient evaluation, emphasizing tailored diagnostic approaches that balance early cancer detection with the avoidance of unnecessary procedures.^6^ Furthermore, recent cost‐effectiveness analyses of microhaematuria thresholds underscore the economic and clinical implications of adjusting diagnostic criteria, particularly in identifying the optimal balance between sensitivity, specificity and healthcare costs.^7^

Current diagnostic methods largely rely on cystoscopy and urine cytology, which are effective but have limitations, including invasiveness for the former and lack of sensitivity for low‐grade tumours for the latter. Due to BC's high recurrence rate, lifelong surveillance is often required, resulting in the highest lifetime treatment costs among cancer patients. This underscores the need for accurate, non‐invasive and cost‐effective diagnostic tools.^8^, ^9^

Cytology‐like and urine‐based genomic and proteomic biomarkers offer promising alternatives. While several tests have been approved by the Food and Drug Administration (FDA), none have yet surpassed cystoscopy and cytology.^10^ These tests include protein‐based urinary biomarker tests such as the NMP22® assay, which identifies subtype 22 of the nuclear matrix protein family. It has been approved under the BladderCheck® test (POC, qualitative) and the ELISA test (quantitative) for the diagnosis and monitoring of BC. Other approved tests are the BTA stat® (qualitative) and the BTA TRAK® (quantitative), which both detect the presence of the complement factor H‐related protein (hCFHrp) in urine by ELISA. The two FDA‐approved cell‐based urinary biomarker tests are Immunocyt (uCyt+), which detects CEA and MAUB biomarkers by immunofluorescence and UroVysion® tests, which detect chromosome aneuploidy using FISH (Fluorescence in situ hybridization).^11^ Inconsistencies in study outcomes and limited validation across diverse cohorts hinder their routine clinical use.

Multicentric trials and clinical studies, conducted simultaneously at multiple medical institutions or sites with common protocols and data collection guidelines, address these gaps by offering significant advantages for the validation of urine‐based biomarkers in BC management. They increase sample sizes and statistical power, improve generalizability by including diverse populations and reduce selection bias. These collaborative studies also centralize expertise and methods, supporting a more comprehensive understanding of BC diagnostic and surveillance needs.^12^, ^13^

This review evaluates multicentre evidence on urinary genomic and proteomic biomarkers for BC detection and surveillance. Additionally, the potential utility of these emerging non‐invasive urine‐based biomarkers in reducing unnecessary cystoscopies among high‐risk populations is also discussed.

## 2 EVIDENCE ACQUISITION

**FIGURE 1:**
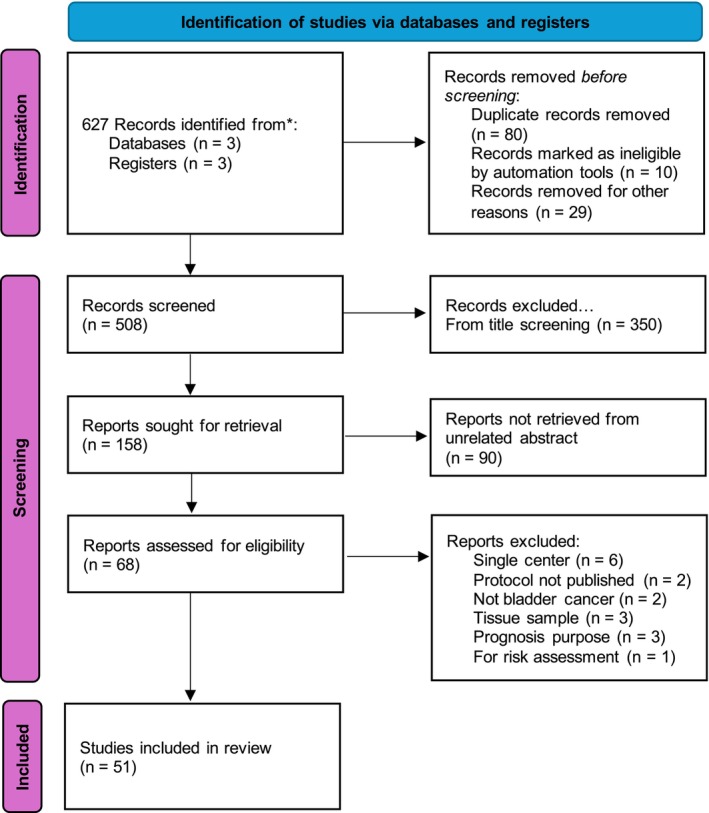
PRISMA diagram.

The study screening methodology is illustrated in the (Preferred Reporting Items for Systematic Reviews and Meta‐Analyses) PRISMA flowchart shown in Figure 1. MEDLINE/Pubmed, Embase and Scopus databases and BJUI, UroToday and European Urology Oncology registries were searched to identify multicentric studies on urinary biomarkers for BC detection published between 1st January 1995 and 1st March 2025. The keywords and MeSH terms used were: (Bladder cancer OR bladder neoplasm OR urothelial carcinoma) AND (biomarker OR protein‐based biomarker OR genomic‐based biomarker) AND urine AND (diagnosis OR surveillance). Articles on multicentric studies for BC prognosis prediction and patient stratification according to risk were excluded from this review. The web search first identified 620 articles and hand search identified 7 articles. The title screening excluded 350 studies and duplicates, and unrelated abstracts resulted in the exclusion of 68 studies. After a further comprehensive review of articles, 51 studies were included based on inclusion and exclusion criteria (Table 1).

**TABLE 1:** Inclusion and Exclusion criteria.

Category	Inclusion criteria	Exclusion criteria
Study type	Multicentric and multi‐institutional studies	Single‐centre studies
Outcome	Evaluation of de detection (diagnosis and/or surveillance) potential of biomarkers	Prognosis prediction, oncological outcomes, risk assessment and therapy
Biomarker type	Urinary biomarkers	Tissue samples
Disease focus	Bladder cancer	Other conditions (prostate, kidney, renal failure, COVID, etc.)
Centre type	Bladder	Any other cancer type
Article type	Original research articles, case reports, short communications	Review article, guidelines, editorials
Biomarker type	Urinary biomarkers	Not urinary

## 3 EVIDENCE SYNTHESIS

Fifty‐one studies were reviewed and summarized in Supplementary Table S1, which describes multicentre and institutional collaborations, study designs, cohort characteristics, methodologies, biomarker types and performance metrics including sensitivity, specificity, area under the curve (AUC) and predictive values. Studies were grouped by the nature of biomarkers in cytology‐like tests, genomic‐based urinary biomarkers and protein‐based biomarkers groups.

### 3.1 Exfoliated cell‐based urinary biomarkers (cytology‐like tests)

Although routinely used, cytology has high false‐negative rates, prompting the development of new urinary‐exfoliated cell‐based tests for improved BC detection and monitoring.^14^

#### 3.1.1 UroVysion ®

The UroVysion® FISH assay, FDA‐approved in 2001, detects chromosomal aneuploidies in urothelial cells and was evaluated in multicentric studies starting in 2002. It showed greater sensitivity and specificity than cytology and the BTA stat® assay for detecting BC recurrence and distinguishing UC from other conditions.^15^ The assay had a sensitivity of 71%, outperforming BTA stat® (50%) and urine cytology (26%) for UC detection, and a specificity of 94.5% against healthy and non‐cancer controls. Later studies reported reduced sensitivity (55.6%) and specificity (67.2%) for recurrence in patients with negative cystoscopy. Despite its promise, variability and false positives limited its clinical adoption.^16^

#### 3.1.2 VisioCyt®

Lebret et al compared the VisioCyt® test and artificial intelligence‐based analysis to cytology for BC detection. Sensitivity was 84.9% vs 43%, higher in high grade (HG) (92.6%) than low grade (LG) tumours (77%), surpassing cytology in both. Specificity was high (81.2%) but lower than cytology (100%). Their study showed that the VisioCyt® algorithm improved sensitivity in detecting urinary‐exfoliated urothelial cells, which could support pathologists in diagnosis.^17^

#### 3.1.3 URO17®

URO17® is another exfoliated cell‐based urinary biomarker evaluated in a multicentric study in 2024 by Ibrahim et al. Overall, URO17® showed 98% sensitivity and 74% specificity, outperforming cytology (83% and 70%). In haematuria patients, it achieved 100% sensitivity and 96.15% specificity vs. 91.3% and 50% for cytology. For recurrence, URO17® showed 85.7% sensitivity and 47.7% specificity, compared to 28.6% and 93.57% for cytology.^18^

#### 3.1.4 CellDetect®

A multicentric study evaluated CellDetect®, a histochemical stain using *Ficus elastica* extract and dyes, for distinguishing normal and cancerous urinary‐exfoliated cells. They compared CellDetect® use with standard cytology and the BTA stat® test. Sensitivities were 84% (CellDetect®), 69% (BTA stat®) and 50% (cytology), while specificities were 70%, 70% and 87%, respectively. CellDetect® showed the greatest improvement in HG‐NMIBC but was outperformed by BTA stat® in MIBC (91% vs. 82%).^19^

### 3.2 Genomics‐based urinary biomarkers

The transformation of normal urothelial cells into a malignant phenotype may arise from genetic and epigenetic alterations, including mutations, DNA and RNA methylation and chromosomal rearrangements. These changes disturb gene expression and signalling pathways, impacting cellular differentiation and proliferation.^20^, ^21^

DNA mutations can be detected in urinary tumour DNA (utDNA), enabling non‐invasive BC diagnosis using cell‐free or exfoliated cell DNA.^22^, ^23^ Multiple multicentre studies evaluated such tests.

#### 3.2.1 Gene mutations biomarkers

##### AssureMDX

Beukers et al evaluated a biomarker panel (*FGFR3* and *TERT* promoter mutations with *OTX1* methylation) for BC surveillance in a multicentric study of 977 NMIBC patients. Sensitivity reached 81% and 94% for LG and HG BC at diagnosis, and 57% and 72% for recurrence. Despite the large cohort, recurrence detection remained limited.^24^ The same group later developed AssureMDX, combining *FGFR3, TERT, OTX1* with *HRAS, ONECUT2* and *TWIST1* for haematuria patients.^25^ The reported sensitivity of 93% for primary detection matched earlier HG patient results. They further evaluated the AssureMDX test, which showed 93% sensitivity, 86% specificity and 99% NPV, again outperforming the NMP22 test and others.^7^ These multicentre studies highlight AssureMDX's high sensitivity and predictive accuracy, supporting its use before cystoscopy. The same group recently evaluated the three‐biomarker panel (*FGFR3*, *TERT* and *OTX1*) for monitoring recurrence in BC in another multicentric study focusing on high‐risk NMIBC patients under surveillance. It resulted in 75% and 70% sensitivity and specificity, respectively, showing that the combination of other biomarkers is a valuable addition to the three previously evaluated.^26^ The same group recently evaluated the three‐biomarker panel for monitoring recurrence in BC in another multicentric study focusing on high‐risk NMIBC patients under surveillance. It resulted in 75% and 70% sensitivity and specificity, respectively, showing improved results for recurrence detection compared to previous work.^26^

##### OncoUrine

The OncoUrine test detects 17 mutations, and one methylation marker was evaluated in a multicentric study. This test achieved a sensitivity of 80% and specificity of 91.9% for primary diagnosis (n = 47) and 100% sensitivity, but a lower specificity of 68.2% for recurrence detection (n = 76). Larger‐scale multicentric studies are needed to validate its efficacy and feasibility.^27^

##### Uromonitor™

The Uromonitor™ test, introduced in 2018, is a non‐invasive qRT‐PCR urine‐based assay for detecting NMIBC recurrence, initially targeting *TERT* promoter and *FGFR3* mutations. The upgraded Uromonitor‐V2® added *KRAS* codon 12 and 61 mutations to enhance performance. In 2019, Batista et al. reported 73.5% sensitivity and 93.2% specificity for recurrence detection, with 100% sensitivity when combined with cystoscopy. Adding *KRAS* mutations raised sensitivity to 100% but lowered specificity to 83.3%.^28^ In 2023, Azawi et al. reported 89.7% sensitivity and 96.2% specificity in pTa‐LG NMIBC patients, surpassing cystoscopy's sensitivity (58%) and reducing false positives. However, the study lacked cytology comparisons and focused on a narrow group.^29^ In contrast, a 2024 study by Wolff et al. found 49% sensitivity and 93.3% specificity, only slightly outperforming cytology. Overall, Uromonitor‐V2® shows potential for NMIBC surveillance, particularly with *KRAS* integration, but is not yet a cystoscopy replacement.^30^

##### UroAmplitude

UroAmplitude is a next‐generation sequencing (NGS)–based urine assay that detects recurrent genomic alterations commonly associated with bladder cancer, including mutations in FGFR3, TERT, PIK3CA and other genes. In a multicentric validation study, the test achieved a sensitivity of 95% and a specificity of 90% for the detection of bladder cancer in patients presenting with haematuria, with a sensitivity of 100% in high‐grade and muscle‐invasive tumours.^31^ In the surveillance setting, it demonstrated 65% sensitivity, 89% specificity and a negative predictive value (NPV) of 91%, supporting its utility for recurrence monitoring. A subsequent investigation confirmed that genomic signatures identified by UroAmplitude were significantly associated with disease recurrence and progression risk.^32^ By quantifying both variant allele frequency and signal amplitude, UroAmplitude provides a more comprehensive genomic profile than targeted PCR‐based assays, offering enhanced analytical sensitivity and reproducibility. Together, these findings highlight its promise as a robust genomic platform for both diagnosis and longitudinal monitoring of urothelial carcinoma within non‐invasive urine‐based workflows.

#### 3.2.2 DNA methylation‐based biomarkers

##### *FGFR3* and *HS3ST2*, *SEPTIN9* and *SLIT2*

This next multicentric study evaluated *FGFR3* mutation and three markers (*HS3ST2*, *SEPTIN9* and *SLIT2*) methylation status at diagnosis and follow‐up points. They integrated all these markers in a single logistic regression analysis and obtained an overall sensitivity of 98% and specificity of 85% for BC diagnosis and sensitivity of 95% and specificity of 76% for BC surveillance.^33^

##### Three‐gene classifier and bladder EpiCheck™

Van der Heijden et al. developed a three‐gene methylation classifier (*CFTR, SALL3, TWIST1*), which achieved a sensitivity and specificity of 85% and 68% respectively for primary diagnosis, with a sensitivity of 91% but a limited specificity of 31% for recurrence detection.^34^ Similarly, the Bladder EpiCheck™ assay analysing 15 DNA methylation markers demonstrated a sensitivity of 68% and specificity of 88% in detecting recurrent BC, with improved sensitivity for high‐grade tumours (92%).^35^ The detection of different DNA methylation markers seems to generate heterogeneous results across different multicentric studies.

##### NID2/TWIST1 and utMeMA

After a previous study showing that *NID2* and *TWIST1* methylation as markers were not superior to urine tests currently in clinics, Hermanns et al identified these markers as independent predictors of BC.^36^ They found that markers achieved sensitivity of 76.2% and 77.6%, with specificity of 83.3% and 61.1%, respectively. Combining markers improved sensitivity but lowered specificity. While promising for detecting BC, the lack of cytology data disabled the possibility of comparing the assay's performance across studies.^37^ In the same year, Chen et al. introduced the utMeMA model, identifying 26 methylation markers with 90% sensitivity and 83% specificity. It outperformed cytology and UroVysion FISH in all cases of BC, especially in small single tumours (81% vs 15% vs 38%), which are harder to detect. However, the model requires further validation for widespread use.^38^

##### T2DMR

The 2022 Challenge‐BLCA study by Xiao et al also developed a DNA methylation assay (*T2DMR*) for BC detection. It showed 100% sensitivity for HG BC, 62% for LG BC and 87.5% overall sensitivity, with 100% specificity. These results outperformed the FISH assay's sensitivity (LG: 28%, HG: 73%) and specificity (80%). While promising, the small sample size (n = 259) requires further validation in larger cohorts.^39^

##### NRN1, GALR1, HAND2

A recent study aimed to evaluate the performance of three methylation markers (*NRN1*, *GALR1* and *HAND2*), which were selected from 11 as being good candidates to diagnose primary BC from urine analysis. The combination of *NRN1/GALR*1 and *NRN1/GALR1/HAND2* markers achieved 84% sensitivity and 96% specificity in a training case–control series, which was further validated in an independent validation at 75%/76% sensitivity and 93% specificity. While the study is small, it showed excellent diagnostic potential for non‐invasive detection of BC, motivating further validation in larger sample sizes.^40^

##### PENK

Jeong et al. compared a *PENK* methylation‐based test to the NMP22 test and urine cytology. PENK showed 78.1% sensitivity and 88.8% specificity, with 89.2% sensitivity for HG or noninvasive BC. In contrast, NMP22 and cytology had lower sensitivities (44.1% and 32.3%) but higher specificities (91.9% and 99.5%). These findings highlight the potential of *PENK* methylation testing as a more sensitive diagnostic tool.^41^

#### 3.2.3 RNA‐based biomarkers

##### uRNA

In 2012, O'Sullivan et al conducted a multicentric study on a urine‐based multigene RNA test analysing *CDC2, HOXA13, MDK* and *IGFBP5* in haematuria patients. The test showed 62% sensitivity and 85% specificity, compared to cytology (56%/95%), NMP22® ELISA (50%/88%) and BladderCheck® (38%/96%). The study also assessed the performance of CxBladder™, which detects the same markers with the addition of *CXCR2* to the panel. This addition improved sensitivity to 82% overall, detecting 97% of high‐grade and 100% of stage ≥ 1 tumours and distinguishing low‐grade pTa tumours with 91% sensitivity and 90% specificity.^42^

##### CxBladder™

A 2017 study evaluated the Cxbladder Monitor's (CxbM) performance to rule out the recurrence of BC. The sensitivity and specificity for intermediate to high risk of progression were 95% compared to 86% for low risk (NMIBC‐LG).^43^ The same group later compared the first‐generation Cxbladder™ test with an enhanced version adding *FGFR3* and *TERT* mutations. In 804 patients, the enhanced Cxbladder‐Detect (CxbD+) tests showed a very high sensitivity of 97% and specificity of 90%, outperforming the original test and many other known urinary biomarker tests. The study also improved risk stratification for haematuria patients (Cxbladder‐triage [CxbT+]), but it lacked cytology data for comparison across biomarker tests and multicentric studies.^44^

##### Xpert® bladder cancer

A multicentric study was first conducted for the development of the Xpert® Bladder Cancer Monitor, a test used for monitoring recurrence in NMIBC via the Cepheid GeneXpert Instrument Systems. After training on multiple mRNA markers, they narrowed down to 5 (*ABL1, ANXA10, CRH, IGF2, UPK1B)*, and a further independent testing revealed a sensitivity of 73% and a specificity of 90% for primary diagnosis in haematuria patients and 77% specificity for recurrence detection.^45^

In a multicentric study by Van Valenberg et al conducted later, the test achieved 74% overall sensitivity (HG: 83% and LG: 63%) and 80% specificity. In comparison, cytology showed much lower sensitivity (30%), but higher specificity (90%), while UroVysion® showed lower sensitivity (51%) and the same specificity. The Xpert® assay was shown to be superior in detecting recurrent tumours compared to cytology and UroVysion®, but its performance did not reach the needed standard for clinical adoption.^46^ A recent article by Abuhasanein et al evaluated those five mRNA markers (referred to as GeneXpert BC) as a potential method for triage of patients presenting with haematuria. The study yielded a high sensitivity (94%) but lower specificity (52%).^47^ Finally, another recent article compared cytology to the Xpert® BC monitor in the detection of CIS, where sensitivity and specificity were 59% and 93% for cytology compared to 90% and 69% for the Xpert® test.^48^

##### IGF2 and CK20

This study measured the levels of IGF2 and CK20 transcripts in urine sediments to evaluate their potential in the detection of BC. The combined analysis of both markers in the Test and Validation cohorts resulted in 78% and 90% sensitivity and 88% and 84% specificity, respectively. When compared to cytology, results were similar (93–95% sensitivity and 72–82% specificity); thus the results are higher than those generally reported in the literature.^49^

#### 3.2.4 MicroRNA biomarkers

In a 2019 study by Piao et al, two urinary microRNAs (*miR‐6124* and *miR‐4511*) showed promise for BC diagnosis, with 91.5% sensitivity and 76.2% specificity. The test's performance was much better than cytology, which showed a very low sensitivity of 7.8% and 25% for LG and HG, respectively. However, this study lacked longitudinal data, emphasizing the need for further research on the recurrence prediction of the test.^50^

### 3.3 Protein‐based urinary biomarkers

As BC‐related changes in DNA and RNA often manifest at the protein level, protein biomarkers have been extensively explored. These include tumour‐specific post‐translational modifications, gene fusion products and abnormal expression levels. Different protein‐based urinary biomarker tests have been evaluated in multicentric settings over the years.^51^

#### 3.3.1 BTA stat®

Sarosdy et al. conducted a multicentric study in 1995 studying the Bard BTA test detecting bladder tumour analytes. The test showed higher sensitivity (40%) than cytology (17%) for recurrent BC, especially in low‐ to mid‐grade tumours.^52^ In 1997, the same group evaluated the improved version called the BTA Stat® test in a multicentric study. The BTA Stat® detects hCFHrp in urine and showed an improved sensitivity of 67% overall for BC recurrence detection, outperforming both the original BTA test and cytology (23%). Despite reduced accuracy in haematuria cases, its superior performance led to FDA approval in 1999.^53^

#### 3.3.2 BTA TRAK**®**

The BTA TRAK® test, which also detects hCFHrp in urine, was evaluated by Thomas et al in a multicentric study in 1999 and approved by the FDA in 2002. It showed 66% sensitivity, outperforming cytology (33%), but had lower specificity (69% vs 99%). The authors noted a high false‐positive rate due to interference from benign genitourinary conditions and haematuria.^54^

#### 3.3.3 AuraTek FDP

In parallel, the AuraTek FDP test, targeting fibrinogen degradation products, demonstrated a sensitivity of 68% compared to 34% for cytology, with notable efficacy in high‐grade tumours.^55^

#### 3.3.4 BC‐116 and BC‐106

Frantzi et al. developed two peptide biomarker panels (BC‐116 and BC‐106) evaluated in a multicentric study showing sensitivities of 91% and 88% and specificities of 68% and 51%, respectively, for BC detection. Despite moderate specificity, both showed value in diagnosis and monitoring.^56^ A 2022 study by the same group confirmed similar sensitivities (89%, 90%) but differing specificities (67%, 29%) for recurrence monitoring. Cytology results were 50% sensitivity and 95% specificity. After combining each panel with cytology, the authors found that BC‐116 alone and BC‐106 combined with cytology could reduce cystoscopy use.^57^

#### 3.3.5 ADXBLADDER test

The ADXBLADDER test detects MCM5 and has been evaluated in several multicentric studies for BC recurrence detection. Rouprêt et al showed an overall sensitivity of 44.9% for recurrence detection in 2020.^58^ Sensitivity and specificity were reported at 52% and 66% respectively by the same group in 2021, with cytology's sensitivity only reaching 17%, despite high specificity (98%).^59^ The group further tested the biomarker specifically for high‐risk recurrent tumours (HG/CIS) in 2022, which revealed an enhanced performance with 67% sensitivity. The authors mentioned the high reliability of the ADXBLADDER test to rule out carcinoma in situ recurrence, which is more likely to be missed by cystoscopy, thus enhancing its clinical utility.^60^

#### 3.3.6 UBC® rapid test

The UBC® Rapid test detects cytokeratin 8 and 18 in urine was evaluated in a multicentric study showing better sensitivity in high‐risk groups (70.8%) but moderate overall specificity (61.4%).^61^ It suits the primary diagnosis better than surveillance. In 2018, Ecke et al evaluated the updated version of the test for BC monitoring performance. They found that the performance was better in high‐risk group, showing sensitivities of 39%, 75% and 68.5% for NMI‐LG, NMI‐HG and MI‐HG groups, respectively, and 94% specificity for all.^62^ Later, the same group compared the performance of multiple urinary biomarker, including BTA stat®, NMP22® BladderChek®, UBC® Rapid Test, CancerCheck UBC® Rapid VISUAL and cytology. Overall, the diagnostic sensitivities for each test were 76.7%, 33.0%, 72.2%, 47.2% and 55.8%, respectively. Specificities were 67.9%, 95.5%, 79.4%, 94.4% and 83.7%. The authors concluded that the BTA stat® and UBC® Rapid Test delivered the best performance in detection of HG NMIBC (83% and 85% respectively) with the highest potential based on their performance, availability and cost‐effectiveness.^63^

#### 3.3.7 Integrin α3β1

Aberrant glycosylation of the integrin α3β1 (AG31) also serves as a biomarker for BC detection via voided urine testing. The large cohort multicentric evaluation of this biomarker resulted in 90.7% sensitivity and 91.52% specificity, and the results were similar between healthy participants and participants with urological conditions other than UC. The group also compared the AG31 test to NMP22, revealing improved performance from AG31 with 91%/98% compared to NMP22 with 447%/87% sensitivity/sensitivity. This biomarker acts as a promising new urinary biomarker for BC detection in urine.^64^

#### 3.3.8 Oncuria™

First, a small study evaluated a panel of 10 proteins (*IL8, MMP9, MMP10, SERPINA1, VEGFA, ANG, CA9, APOE, SDC1* and *SERPINE1*) to detect recurrent BC. The combined assay reached 79% sensitivity and 88% specificity.^65^ A larger multicentric study evaluated the same panel of proteins, resulting in an overall sensitivity of 79% and specificity of 79%.^66^

The Oncuria™ detects the presence of almost the same panel of 10 proteins in urine (*A1AT, APOE, ANG, CA9, IL8, MMP9, MMP10, PAI1, SDC1* and *VEGFA*) and was evaluated in multiple multicentric studies. A multicentric study evaluated this test, and the overall sensitivity and specificity were 93% and 93% respectively. The assay is currently being tested in even larger multicentric and international studies, which will improve the accuracy of its diagnostic performance.^67^

## 4 DISCUSSISON

The landscape of urinary biomarkers for bladder cancer is rapidly advancing, but significant hurdles remain before they can be integrated into routine practice as an alternative or adjunct to cystoscopy. The multicentric studies reviewed here provide important insights into both the opportunities and limitations of current approaches.

### 4.1 Clinical context of haematuria and biomarker use

Haematuria is the most frequent presenting symptom of BC, yet its non‐specificity leads to a high number of unnecessary cystoscopies.^68^, ^69^ Robust urinary biomarkers could serve as triage tools, identifying which patients with haematuria require invasive evaluation while sparing others. Importantly, a subset of patients develops BC in the absence of haematuria, and biomarker‐based screening in high‐risk groups—such as smokers and individuals with occupational exposures—could facilitate earlier detection of asymptomatic disease.

### 4.2 Biological and technical considerations

Different biomarker classes reflect complementary aspects of tumour biology. Genomic and epigenomic markers capture early oncogenic events, while protein‐based biomarkers often mirror tumour burden, immune responses or microenvironmental changes. Both categories, however, are influenced by confounding conditions such as infection or inflammation, which can lower specificity. Integrating multiple biomarker classes into composite panels, or combining them with cytology, may help balance sensitivity and specificity. Such multiparametric approaches also offer opportunities to gain mechanistic insights into tumour progression and treatment responses.

### 4.3 The need for non‐invasive biomarkers in bladder cancer

Developing non‐invasive biomarkers for BC is crucial due to the limitations of current diagnostic and surveillance methods. Cystoscopy, the gold standard, is highly sensitive but invasive, uncomfortable and costly. Commercially available kits provide a streamlined approach for biomarker testing, but face adoption challenges due to cost, variable performance and lack of widespread clinical validation. Given the financial burden of repeated cystoscopies, especially in patients with recurrent haematuria or BC, there is a pressing need for reliable, cost‐effective biomarkers that enhance care and quality of life while reducing healthcare costs.^70^ Non‐invasive tests could also enable screening in asymptomatic individuals, especially high‐risk groups such as smokers, workers exposed to bladder carcinogens and possibly other populations at risk of developing BC. To be effective in these groups, biomarkers should exhibit very high specificity and positive predictive value (PPV) to minimize false positives and avoid overwhelming healthcare systems with unnecessary cystoscopies. In parallel, the test should also meet cost‐effectiveness criteria by significantly reducing the number of cystoscopies in subjects with negative tests, thereby reducing overall costs associated with BC management.^71^, ^72^

From this current review of urinary biomarkers evaluated in multicentric studies, AssureMDX and the enhanced CxBladder tests stood out based on sample size, study design and detection performance. AssureMDX has been assessed in two independent multicentric studies for primary BC diagnosis, with its surveillance value yet to be explored. Enhanced CxBladder, combining six SNPs in the FGFR3 and TERT genes with five established mRNA biomarkers, showed notable improvements over the standard version. These results suggest that both tests may be among the most effective non‐invasive tools currently available for the diagnosis and monitoring of BC. Other promising tests, like OncoUrine and T2DMR, show potential but were limited by smaller sample sizes, and Uromonitor, showed variability across studies.

Genomic biomarkers, such as DNA mutations, methylation changes and urinary miRNAs, show strong diagnostic potential, especially when combined. Multicentric studies support these findings, though larger cohorts are still needed to capture BC heterogeneity. Practical challenges remain, such as managing large marker panels and accounting for treatment effects like BCG‐induced methylation changes.^73^ Protein‐based biomarkers also show promise. While some offer moderate performance alone, combining them with cytology or integrating them into newer tests like BC‐106 and BC‐116 enhances accuracy. Such combinations may optimize BC detection and surveillance, offering cost‐effectiveness and easy use, ultimately reducing dependence on invasive procedures and improving patient care.

### 4.4 Lessons from multicentric studies

Despite FDA approvals for several assays, no urinary biomarker has replaced cystoscopy in daily practice. Barriers include inconsistent performance across tumour stages, absence of definitive cost‐effectiveness analyses, limited reimbursement and lack of prospective randomized trials demonstrating clinical benefit. Physician confidence and patient acceptance, though generally favourable for non‐invasive tests, also require systematic evaluation. Two recent systematic reviews have advanced the evaluation of urinary biomarkers for BC detection. A 2021 meta‐analysis by Laukhtina et al. compared five promising novel tests, the ADXBLADDER, Xpert Bladder, Cxbladder, Uromonitor and Bladder EpiCheck, for NMIBC diagnosis and recurrence. All five tests outperformed cytology, with Uromonitor leading, but further multicentre validation was advised before stating any specific recommendations. Similarly, Papavasiliou et al. reviewed urinary biomarkers in the context of primary care, highlighting variability in test performance and the importance of validation in larger, multicentre and community‐based studies.^74^, ^75^

Multicentric studies play a vital role in validating biomarker robustness. By applying standardized protocols across diverse populations and clinical practices, they produce more generalizable results. While meta‐analyses offer strong statistical power, multicentric trials provide more meaningful, real‐world insights by accounting for variability across different centres. For example, peptide panels like BC‐116 and BC‐106 showed consistent results in multicentric settings, though variability in specificity emphasizes the importance of standardization and clinical context.^12^ This review, focusing on multicentric studies and FDA‐approved or promising biomarkers, highlights both progress and ongoing challenges in BC diagnostics and surveillance.

### 4.5 Future directions

Future progress will likely depend on the integration of different biomarker classes—genomic, epigenomic, proteomic and cytology‐based—into composite panels that leverage the strengths of each approach. Advances in artificial intelligence may further enhance the sensitivity of cytology‐like methods, offering automated support to pathologists and reducing inter‐observer variability. Biomarkers will also need to be incorporated into risk‐stratified surveillance algorithms that combine molecular results with clinical risk factors and longitudinal data to individualize cystoscopy frequency. In parallel, pragmatic multicentric clinical trials embedded in real‐world practice are essential to determine whether biomarkers can safely reduce cystoscopy use while maintaining oncologic outcomes. Finally, mechanistic studies are required to clarify how treatments such as BCG alter biomarker profiles, ensuring accurate test interpretation in treated patients.

Taken together, urinary biomarkers hold strong promise to reduce invasive procedures, lower healthcare costs and improve patient quality of life. However, their full clinical utility will only be realized through rigorous multicentric validation, methodological standardization and demonstration of cost‐effectiveness within prospective trials.

## AUTHOR CONTRIBUTIONS

A.M., L.R. and P.A. contributed equally to the conception, design and drafting of the manuscript. A.R. and T.E. contributed expertise on clinical urology and critically revised the sections related to diagnostic performance and clinical application. J.R. generated the supplementary Table S1 comparing all the known tests evaluated in multicentric studies. P.A. and F.L.C.‐K. provided input on biomarker development and validation within multicentric frameworks and revised the manuscript for accuracy and scientific integrity. C.J. and E.V. contributed clinical insight and contextualized the role of biomarkers. F.‐M.B. edited the final draft and is the corresponding author. All authors revised the manuscript critically for intellectual content and approved the final version.

## CONFLICT OF INTEREST STATEMENT

The authors declare no conflicts of interest.

## Supporting information


**Table S1.** Supporting Information.
